# Assessment of pavement deflection under vehicle loads using a 3D-DIC system in the field

**DOI:** 10.1038/s41598-022-13176-3

**Published:** 2022-06-08

**Authors:** Carlos Núñez-Temes, Guillermo Bastos, Marcos Arza-García, Alberte Castro, Jose Antonio Lorenzana Fernández, Juan Ortiz-Sanz, María Portela, Mariluz Gil-Docampo, Francisco Javier Prego

**Affiliations:** 1 CIGEO – Civil and Geomatics Research Group, Agroforestry Engineering Department, University of Santiago de Compostela, Higher Polytechnic School (Lugo), 27002 Lugo, Spain; 2 PEMADE – Research Platform on Structural Wood Engineering, Agroforestry Engineering Department, University of Santiago de Compostela, Higher Polytechnic School (Lugo), 27002 Lugo, Spain; 3 Department of R+D+I of Misturas, S.A., Orense, Spain

**Keywords:** Engineering, Civil engineering

## Abstract

This study aims to introduce the use of 3D-digital image correlation (DIC) to the in situ testing of pavements and to support the development of techniques for a rapid evaluation of the conservation status of existing roads. Little research was found on this topic. The passage of a car wheel on an asphalt pavement was adopted as a case study. The DIC measurements were compared to those gathered by contact sensors. From a qualitative point of view, the DIC measurements captured the realistic shape of a deflection basin. From a quantitative point of view, the deflection values provided by the DIC system had a mean error of 0.015 mm and a standard deviation of 0.011 mm. At the moment of highest load, these errors had a mean value and standard deviation of − 0.016 mm and 0.021 mm, respectively. Thus, to improve the accuracy of the system, we propose modifying the camera support, speckle pattern, and control of natural light.

## Introduction

Roads are key public infrastructure since they constitute a basic requirement for the social and economic development of any country^1^. Roads require preservation, maintenance, repair and rehabilitation^2,3^. To plan and perform these actions, reliable information is needed about the status of preservation of roads.

The challenging aspect of road assessment lies in its structural state. The traditional techniques for studying the mechanical properties of bituminous pavements and concrete consist of laboratory testing of samples by using extensometers, strain gauges, and linear-variable differential transformer sensors to measure sample deformation^4–7^.

The in situ modulus and critical stresses and strains of pavements have been determined by measuring the vertical deflection of pavement with falling weight deflectometers since the 1970s^8^ and fast falling weight deflectometers since 2015^9^. The present paper describes deflection measurements of asphalt pavement using the digital image correlation (DIC) technique to explore the feasibility of performing tests in the field with the accuracy of laboratory techniques. Our objectives were to explore the introduction of DIC technology for testing pavement in the field and, in particular, to accurately measure the deformation of asphalt pavement through 3D-DIC (also known as stereo-DIC).

### Background on testing pavement in the field

Several in situ nondestructive testing procedures for pavement are available. The main principle consists of applying a load of known value to the pavement and analyzing the direction and size of the induced deformation^10^. The most common deformation is in the vertical direction^11–13^.

In field tests, there are three typical methods of deflection measurement: static load-based deflectometer, which provides the maximum deflection under a static load^14^; stationary dynamic impact deflection measurement, as the falling weight deflectometer (FWD)^15^; and moving dynamic deflection measurement, e.g. the laser dynamic deflectometer (LDD), which captures the deflection along a line on the pavement as the vehicule moves^16,17^. Though the static load-based Benkelman beam has been used extensively in India and several other countries for structural evaluation of in-service pavements, the FWD is considered to be the most appropriate equipment^14,18^.

Methods applying stationary dynamic loading allow the measurement of the deflection basin under steady-state loads or impacts. This type of equipment consists of an oscillating force generator, a calibration unit and several deflection-measuring devices (transducers, accelerometers, seismometers, etc.)^19,20^. The FWD is the accepted worldwide standard^15,21^. It is mounted in a vehicle that must be stationary to perform a test at the desired location^20^. The FWD imparts a dynamic load to a pavement structure; the dynamic load is similar in magnitude and duration to that of a moving wheel load, typically 50 kN^22,23^, and ranging from 7 to 150 kN for the standard FWD^24^. A light version (LWD) applies 1–15 kN and various heavy versions (HWD) apply up to 250 kN^25^. The peak deflections at each test location are measured in micrometers. First introduced in Europe, FWD has been in use in the United States since the 1980s^21^. The U.S. Federal Highway Administration website provides information about the most commonly used steady-state devices and FWDs^19^. FWDs use a realistic simulation of actual wheel loading and have the ability to measure load transfer across joints and cracks^26^.

However, FWDs also have drawbacks; their stationary operating mode limits the coverage of a test site in a given time, and interruptions due to stop-and-go operations can disturb traffic and cause hazardous conditions. To continuously measure pavement deflections at higher speeds, extensive efforts have been carried out over the past decades to develop methods using moving vehicular loads and continuous profiling devices^15^. Some of both stationary dynamic loading^27^ and moving dynamic loading devices^15^ adopted the test principle of the Benkelman beam test. Some examples are the traffic speed deflectometer (TSD)^28,29^, rolling weight deflectometer (RWD)^30,31^, rolling dynamic deflectometer (RDD)^32,33^, and road deflectometer (RDT)^34^. Elseifi et al.^35^ compared these devices and found that the RDT and the RWD can acquire data while moving at 96.6 km/h with deflection accuracies of 0.25 mm and 0.064 mm, respectively.

Currently, new measurement techniques are gaining momentum for characterizing heterogeneous materials^36^. In particular, DIC systems are increasingly used in testing in civil engineering laboratories^37^ and for determining asphalt microstructure^38,39^, studying crack initiation and damage distribution^40,41^, and validating models^42,43^.

### Introduction to DIC

As the requirements for information about a specimen increase, the large-scale deployment of wired strain gauges becomes more laborious and expensive. In addition, strain gauges are susceptible to drift and damage^44^. To address these limitations, DIC was introduced in the 1980s as a contactless sensing method that records grayscale digital images during the loading of a specimen and applies image processing techniques to estimate the full-field deformation of the specimen^45^.

The basic operation in DIC involves tracking points (or pixels) in consecutive photos. Heterogeneous materials can have surface features that suffice as a natural pattern that facilitates this tracking. However, an artificial pattern, called a speckle pattern, is usually applied to the surface to facilitate image correlation. For each pair of images, matching is performed between subsets of the speckle pattern. The subsets are usually square but can also be conformal, in order that geometries capture the outline of a particular part or region^46^. Two key parameters in a DIC calculation are the subset size and step size^47^. The subset size is critical to the accuracy of the computed displacements^48^. After the full-field displacements are evaluated, spatial strains are generally computed from the displacements with a spatial derivative^49,50^.

Since the introduction of the first DIC technology, the quality of the digital images and processing algorithms have improved enormously. Due to its flexibility and a very simple measurement setup (two synchronized cameras and lighting), DIC techniques are applied widely, not only in the field of engineering but also in medicine, multimedia, conservation of cultural heritage, etc.^51^. Currently, DIC methods with high sensitivity and accuracy are utilized for testing specimens and, most recently, for testing whole structures. It is possible to obtain sub-pixel accuracy of DIC measurements by means of interpolation of image intensity between pixels. Most commercial DIC packages integrate optimized interpolants, allowing to detect image variations up to 0.02 pixel. Depending on the scene configuration, this resolution level could even reach sub-micron accuracies^52^.

Researchers have proved the utility of DIC for measuring deformations in laboratory testing of wood^53^, concrete^54^, masonry^55^, glass panels of curtain walls^56^, and composites^57^, etc. Measurements using DIC in the field are becoming more widespread, including applications in metal additive manufacturing^58,59^, welding-induced deformations^60^, trees^61,62^, thermal barrier coatings^63^, and aircraft crashes^64^, from the micro- to the structural scale^65^. Several in situ experiments were carried out also in civil engineering on concrete bridges^66^; other large structures^67^; diaphragm walls, which are used as a support for deep excavations^68^; and displacements and strains of pipelines due to temperature variations of the fluid^68^.

Several authors have discussed the attractiveness of DIC for testing pavements. In fact, this technique has been applied successfully in the laboratory in compression tests^69,70^, fatigue tests^71^, shear tests^72^ and indirect tensile tests^73,74^. However, few studies have been performed in the field^37^. DIC has already been tested on moving vehicles by Shoop et al.^75^ and in structural monitoring. These authors used DIC to capture the roughness of terrain at the millimeter scale, as their objective was to improve the maneuverability of vehicles by monitoring the road or terrain surface before and after a tire passes over it. The high spatial resolution achievable with DIC, could allow to measure the pavement deflection induced by conventional vehicle loads. This would represent a key advantage of DIC over other major pavement assessment techniques mentioned above, and would make it possible to dispense with the use of heavy towed equipment. Nevertheless, this technology is not yet being used to its highest potential as a reliable and flexible method for measuring displacements and strains at high spatial resolution in the field^76^.

## Materials and methods

This study examined a pavement built near the asphalt plant owned by the paving company Extraco S.A. in northwest Spain (42.204193° latitude and − 7.791827° longitude), in a consortium of three more private organizations and two university research groups. The pavement was built as an experimental stretch of 25 m long and 3.5 m wide, with the geometry of a conventional road lane.

**Figure 1:**
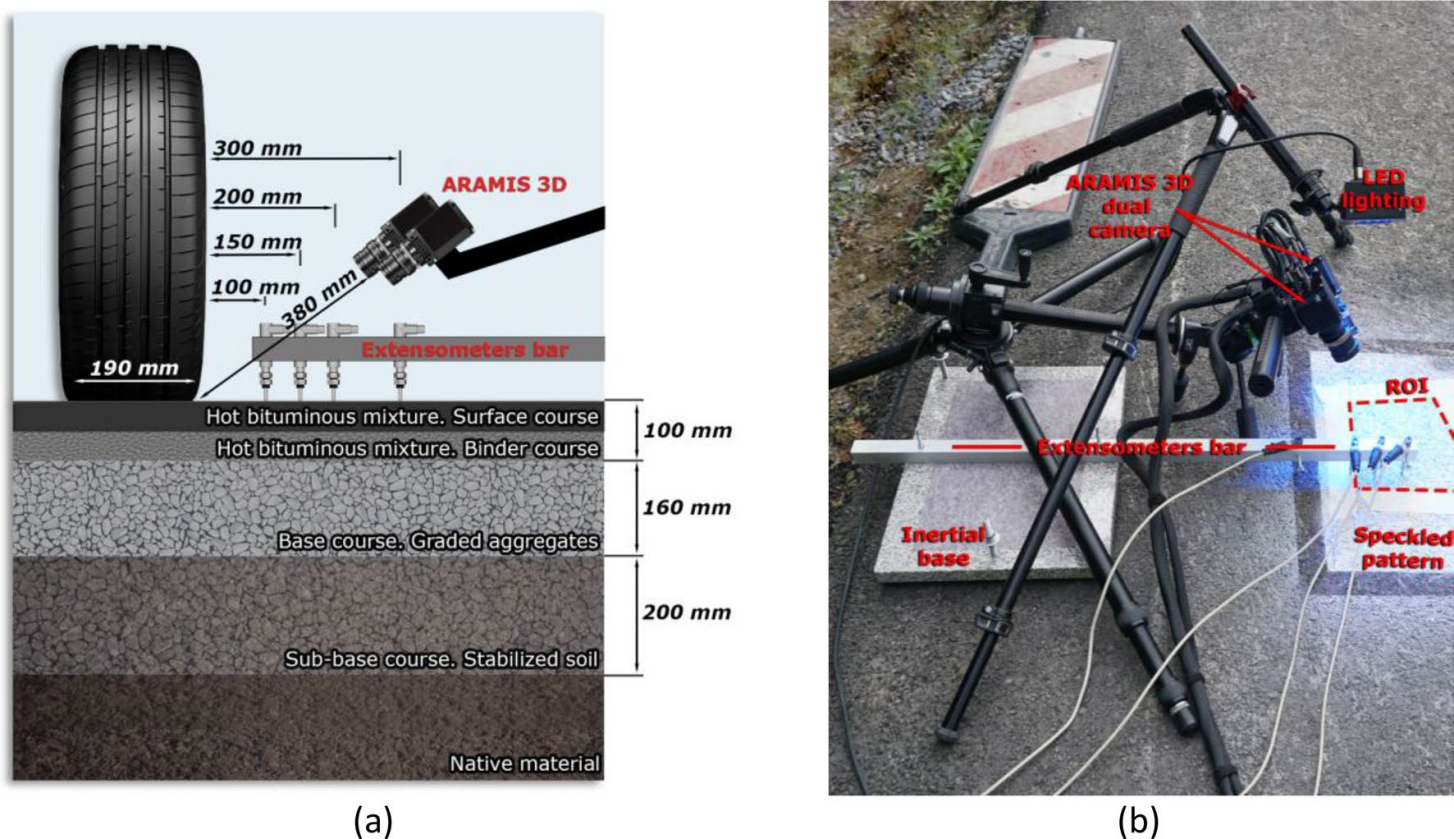
Experimental setup: (**a**) diagram of pavement structure and data acquisition systems, and (**b**) overall setup of the equipment used to monitor the wheel load.

The pavement section adopted (Fig. 1a) consists of a 5 cm wearing course of hot bituminous mix AC 16 surf 50/70 D (according to the Spanish standard UNE-EN 13108-1:2019) with a bitumen content of 5.0% and with 0.5% of the additive rich in lignin, a 5 cm intermediate layer with a hot bituminous mix AC 22 surf 50/70 D with a bitumen content of 5.0% and without additives, and a 16 cm base layer based on artificial gravel ZA 0/32. The sub-base was made up of a 20 cm layer of soil stabilized in situ with also 2% of the lignin-rich additive. A geotextile separates the built layers from the natural ground. The co-product rich in lignin from the eucalyptus wood panel industry^77^ was added in the hot bituminous mix, aiming to reduce the proportion of binder. The object of study of that project was to know how this additive affects the workability of the mix, the construction of the pavement and its long-term behavior.

A speckle pattern was sprayed on the monitored region of interest of the pavement, using a fine aerosol white coating followed by a spot distribution of black paint (25–40% of coverage). The DIC equipment was the commercial Aramis 3D system by GOM GmbH (Braunschweig, Germany), with dual 12 M rev03 cameras and Titanar B 24-mm lenses. As shown in Fig. 1b, the DIC cameras were mounted on tripods. The cameras have 12-megapixel resolution and a maximum image capturing rate of 25 fps at maximum resolution^78^, and the angle between their optical axes was 25.2°.

**Figure 2:**
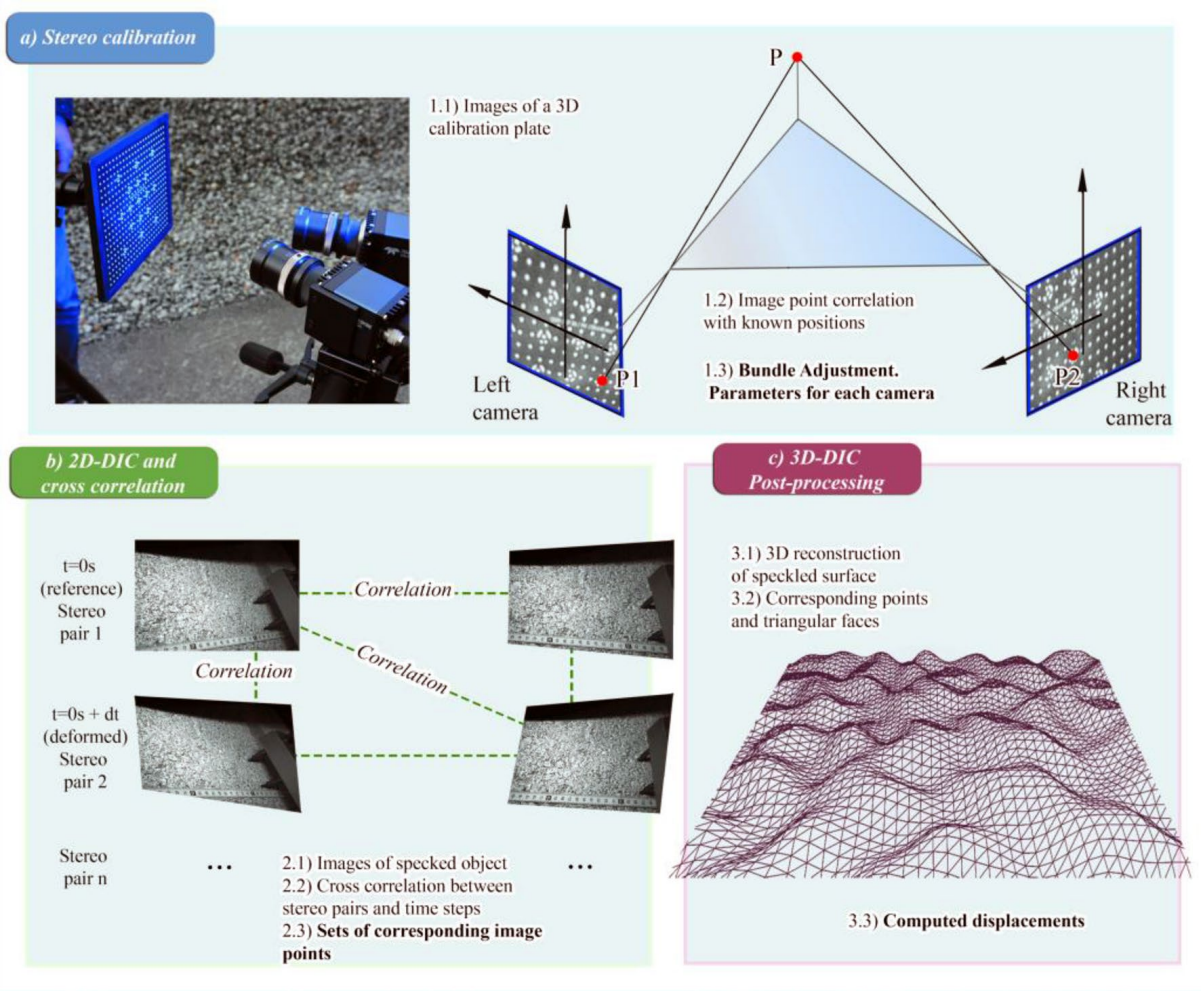
Workflow with the core steps in stereo-DIC processing: (**a**) Stereo calibration procedure to find intrinsic and extrinsic parameters of the optical system; (**b**) cross-correlated 2D-DIC and (**c**) 3D-DIC post-processing to calculate 3D coordinates of the triangular mesh’s vertices and to derive the full-field displacements. Adapted from Arza et al.^82^.

The first stage in the general workflow of stereo-DIC (Fig. 2) is the system calibration (required for stereo triangulation). For each camera of a typical stereo-pair, calibration aims to find the intrinsic parameters (defining the geometric and optical characteristics of the camera) and the extrinsic parameters (defining the position and the orientation of the camera with respect to a reference coordinate system). These parameters can be calculated through the comparison of optically measured deformations and theoretical predictions, for which images of a calibration plate with known dimensions are normally used. In particular, Aramis 3D uses its own in-house calibration plate with a regular grid of dots and auto-detectable coded targets and a specific calibration routine based on the Bundle Adjustment (BA) method. The user is required to take a series of stereo pairs of images by varying the position of the plate (rotations and translations). BA allows for the estimation of both the intrinsic and extrinsic camera parameters by using these repetitive observations of sparse scene points in those different viewing directions^79,80^. Together, the intrinsic and extrinsic parameters serve to describe the transformation that maps each 3D material point P in the global coordinate system into its image point on the camera sensor^81^. As these parameters (camera poses) define for themselves the relationships between the image space and the 3D space, there is some flexibility with regards to calibrating the cameras outside of the actual experimental setup. For this reason, the cameras can first be calibrated in a horizontal position (Fig. 2a) and oriented towards the pavement after completing the procedure.

The second stage of DIC processing consists in tracking the speckled pattern in the sequence of stereo-images of the surface during the test. This process involves multiple 2D-DIC correlation runs, including cross-pair and cross-camera subset matching, where the image from one camera is the reference image and the image from the other camera is the deformed image. During this process, the software correlates homologous points along the whole dataset, identifying their coordinates in each of the images. Then, the calibration parameters from stage 1 are used to perform a stereo-triangulation which transforms the 2D points matched in stage 2 for each stereo-pair into point clouds. In order to obtain a more efficient 3D data analysis, point clouds are converted to triangular meshes. The surface displacements along the test can finally be obtained by using 3D surface comparison algorithms.

Data was acquired on a sunny and windless day, with temperatures ranging from 17.1 to 23.6 °C, falling within the recommended range of DIC operation^83^. The major problems with the temperature in indoor DIC experiments usually occur because almost all cameras and lights become hotter than room temperature when run continuously. Outdoors, this problem should be in part minimized, but instead, other relevant issues that could introduce errors in DIC results may arise. Some of these potential issues are the thermal expansion of the components of the cameras, the lenses, or the mounting structures and the induced convective air currents. In that sense, several precautions have been taken to prevent possible errors. The cameras and other equipment were not calibrated or used to take measurements until they have reached a stable operating temperature in the field (at least 15 min). On the other hand, the lighting required for DIC may introduce heat waves that could refract light between the test surface and the imaging system. A special “cool” (blue) LED source with an integrated fan was used (Fig. 1b) to minimize this effect.

**Figure 3:**
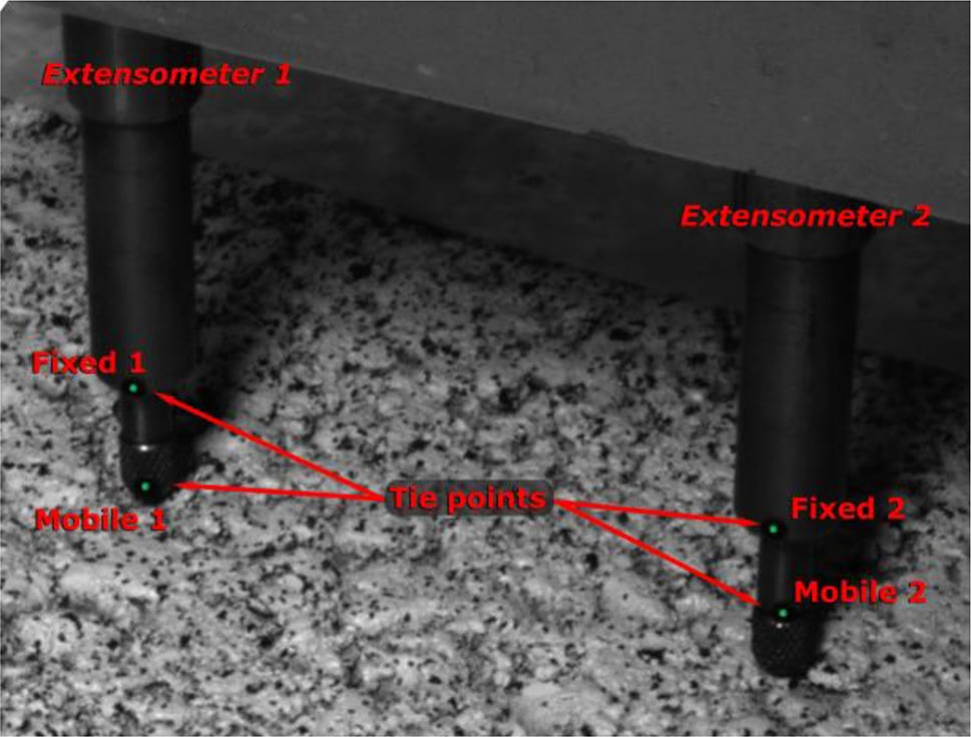
GOM's auto-detectable targets (tie points) attached to the extensometers.

We also recorded the deflection values through four 1-µm-precision extensometers, which we adopted as the reference. In addition, four reference point markers (tie points) were attached to these sensors to read their displacement according to the DIC device, as presented in Fig. 3. Extensometer 1 was closest to the loaded area. The comparison of the DIC readings at these points with the readings provided by the extensometers allowed a direct assessment of the agreement between the DIC device and the contact sensors.

The minimum distance between the middle point between the camera lenses and the loaded ground was 380 mm. The measured area had a width of 195 mm and a maximum length of 205 mm approximately, which decreased during the test to the minimum length of 145 mm, because the wheel partially obstructs the view of the cameras. The extensometers were attached to a square aluminum beam, and they were lowered until they made contact with the asphalt pavement.

The loading wheel was initially placed 2280 mm from the DIC-monitored area. The pavement was loaded by a pass of a moving car, more specifically through one of its wheels, with a load of approximately 3.8 kN. The tire covered an approximately rectangular area of 135 mm in the direction of motion and 190 mm in the orthogonal direction. The pass of the wheel started after the contact sensors stabilized.

## Results and discussion

After the sensing equipment was turned on and the extensometers stabilized, the vehicle moved at approximately 78 mm/s. The DIC system acquired the images with a shutter speed of 1/500 s and at a frequency of 0.5 images per second, resulting in a total of 21 images. Therefore, in terms of DIC processing, we consider 20 time “steps”, as the software matches the pattern between the reference image (first) and the other ones, computing the displacements of the pattern in each one of these moments.

**Figure 4:**
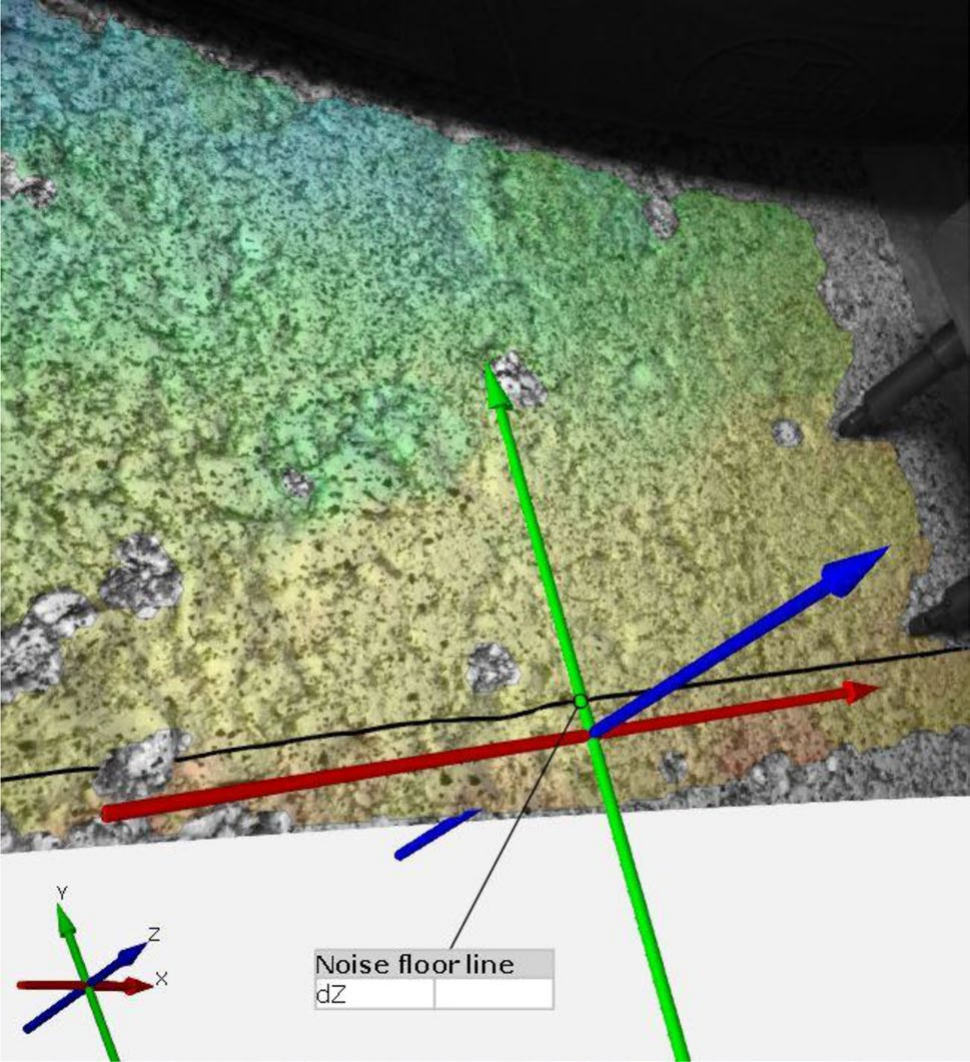
The line across which the noise-floor was evaluated.

**Figure 5:**
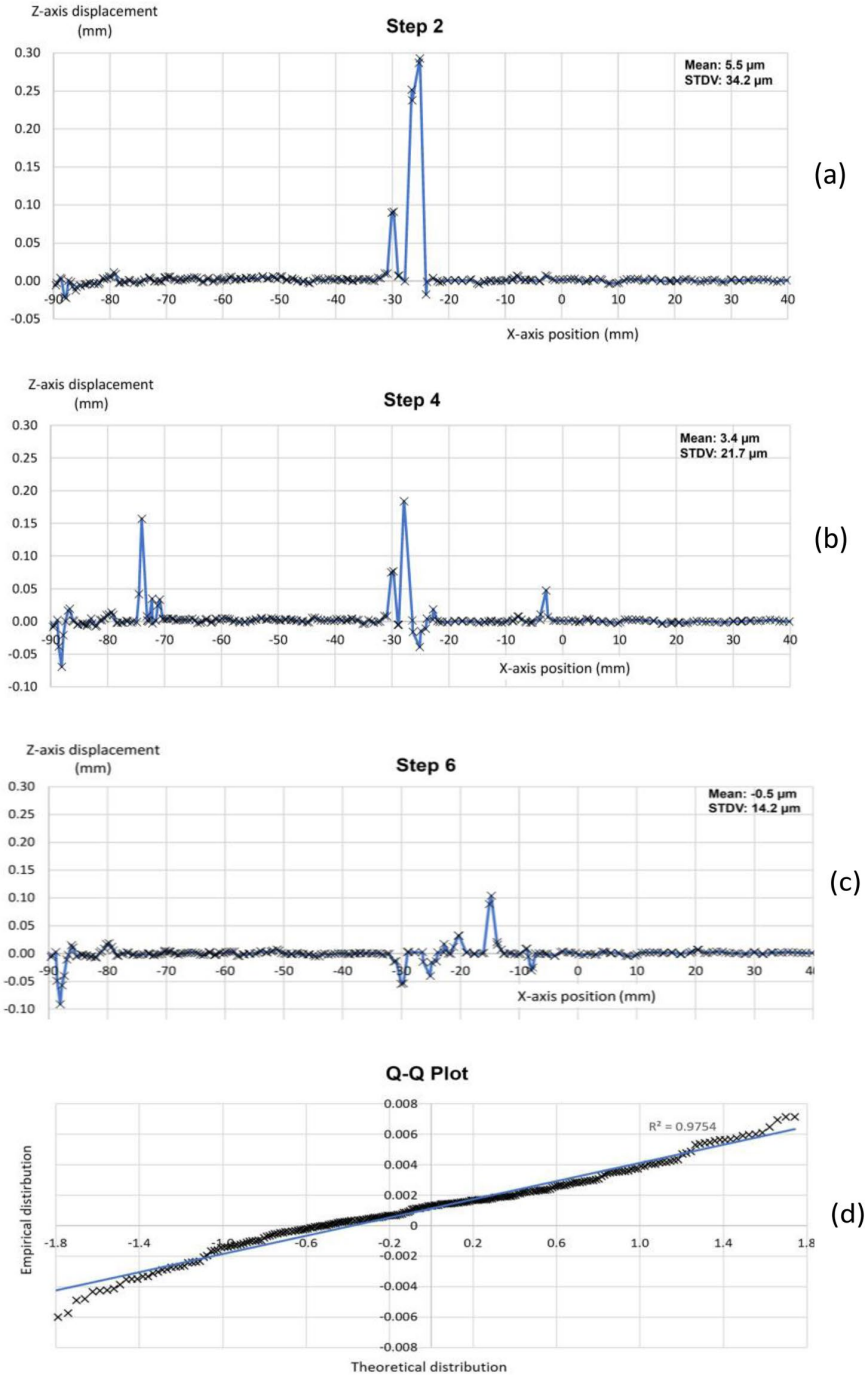
Noise baseline computed from DIC processing. Sample of initial steps not affected by the wheel load: (**a**) Step 2; (**b**) Step 4; (**c**) Step 6; (**d**) the Q-Q plot for step 2.

The noise-floor was measured across the vertical deformation along a line parallel to the wheel motion (see Fig. 4). This noise is a multiple of the spatial standard deviation of the quantities-of-interest (QOI) computed under conditions in which the QOI should be zero. It does not reflect any systematic bias errors that may be present in the QOI, but only the random variance error of the QOI^83^. In this case, the QOI is the deflection. We obtained the noise-floor by taking the first 9 steps. They are considered quasi-stationary, what is corroborated not only by the DIC images, but also by the readings of the extensometers. These readings indicate that significant deflection (> 0.01 mm) are only detectable from step 12 onwards. A representative sample of the noise-floor is presented for three steps in Fig. 5a–c. In step 6 (Fig. 5c), the wheel axle was at 1728 mm on the X axis. In these graphs, the deformation reached a positive peak of 0.29 mm and a negative peak of − 0.09 mm. The Q-Q plot (Fig. 5d) of these data reveals that they fit a normal distribution. The z-score of these extreme values ranged from 2.4 to 8.4. We hypothesize that this was the result of frame mismatching due to the potential poor quality of the speckle pattern in some specific areas. In fact, during the unloaded steps, the deflection in some of these areas alternated between positive and negative values, as shown in Fig. 5. These anomalous data were avoided by applying in the DIC software a median filter of three points to the values of displacement. In these initial steps, the detected deflections are not related to the pavement load.

With regard to the noise in the detection of the tie points (auto-detectable targets), in the four first Steps, when the car wheel reached a distance to the sensors of 1.85 m, the reported values had a mean of absolute values of 1.2 µm and a standard deviation of 1.1 µm. The maximum absolute value was 3.3 µm, which is the 4.3% of the maximum value reported.

An adequate balance between exposure time and the moving velocity is essential to limit motion blur in DIC. The noise-floor can be considered the most conservative estimate for the maximum allowable object motion over the course of the exposure time^83^. In this case, the estimated displacement per exposure is almost negligible (~ 0.4 µm) and much lower, in any case, than the noise-floor (considering a very conservative estimate of the displacement velocity of the points on speckled pattern (~ 0.2 mm/s) and the exposure time employed (1/500 s)).

**Figure 6:**
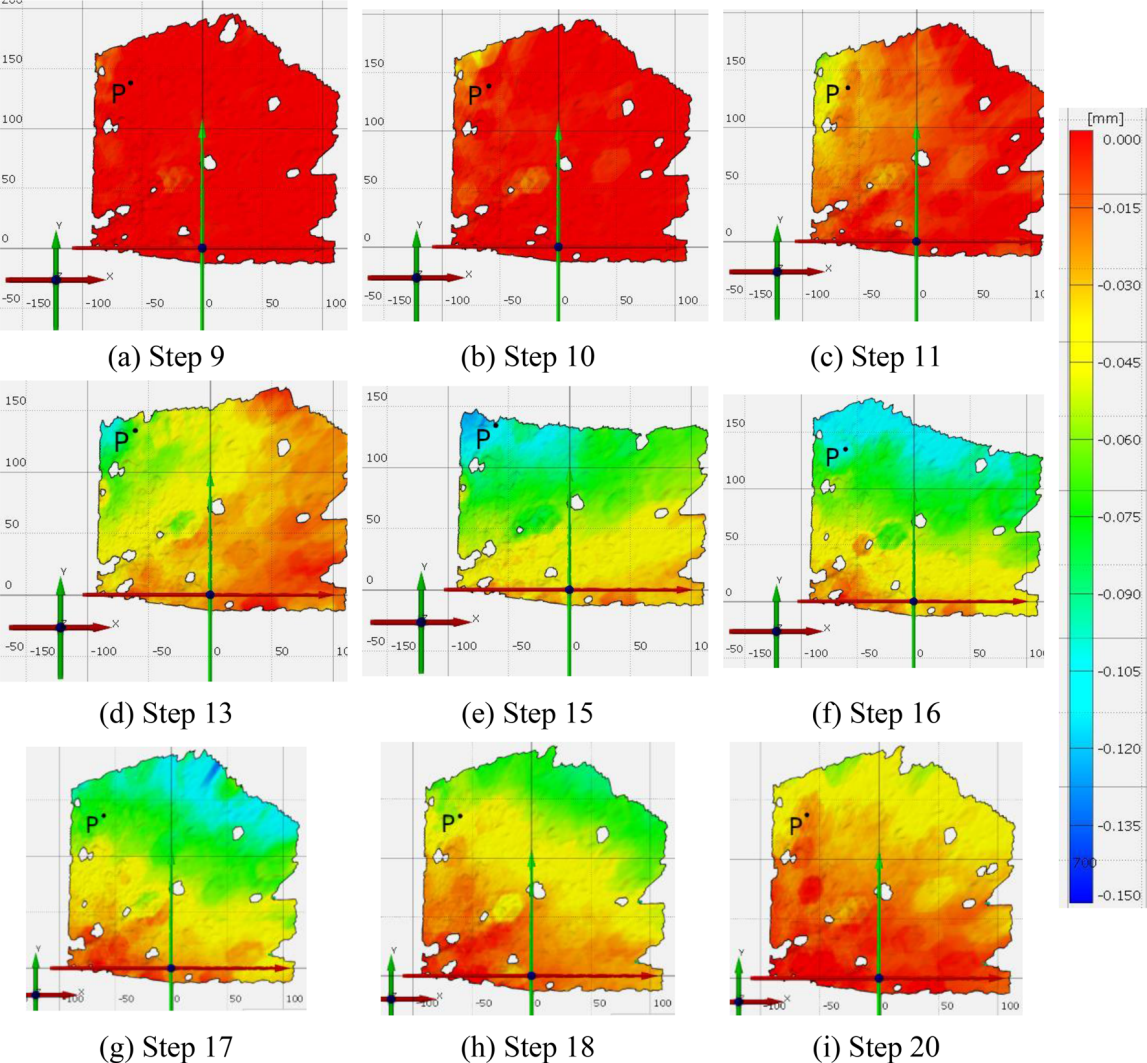
Representative deflection conditions captured by the DIC system. The direction of wheel motion is the X-axis and the border of the pavement contact area coincides approximately with Y = 160 mm. The point P is the closes point in which measures were recorded in all steps.

A sample of representative load conditions is presented in Fig. 6a–i. The captured deformation becomes more evident in step 11, as it can be seen in Fig. 6c. In view of the images of step 13 and step 16, the cameras captured not only the pavement next to the tire but also part of the area behind and front of the tire, respectively.

**Figure 7:**
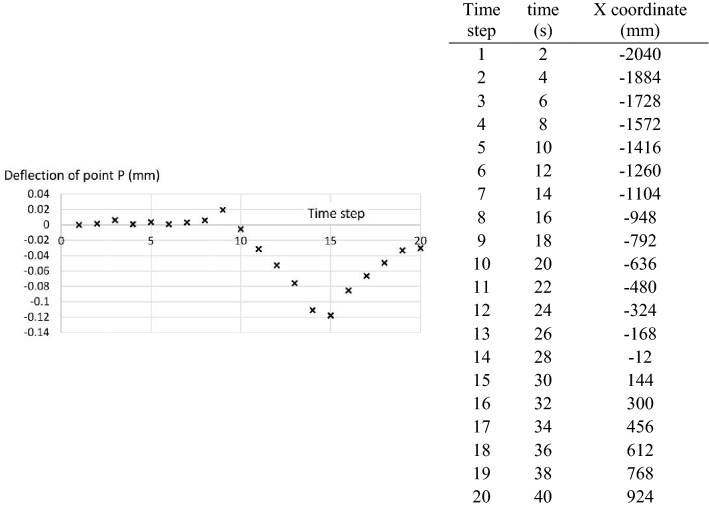
Variation with time of: (left) the deflection at point P; (right) the center point of the loaded area. The X axis is represented in Fig. 4.

The variation with time of the point P (introduced in Fig. 6) is shown in Fig. 7. This figure also contains the position of the wheel over the test, through its X coordinate.

**Figure 8:**
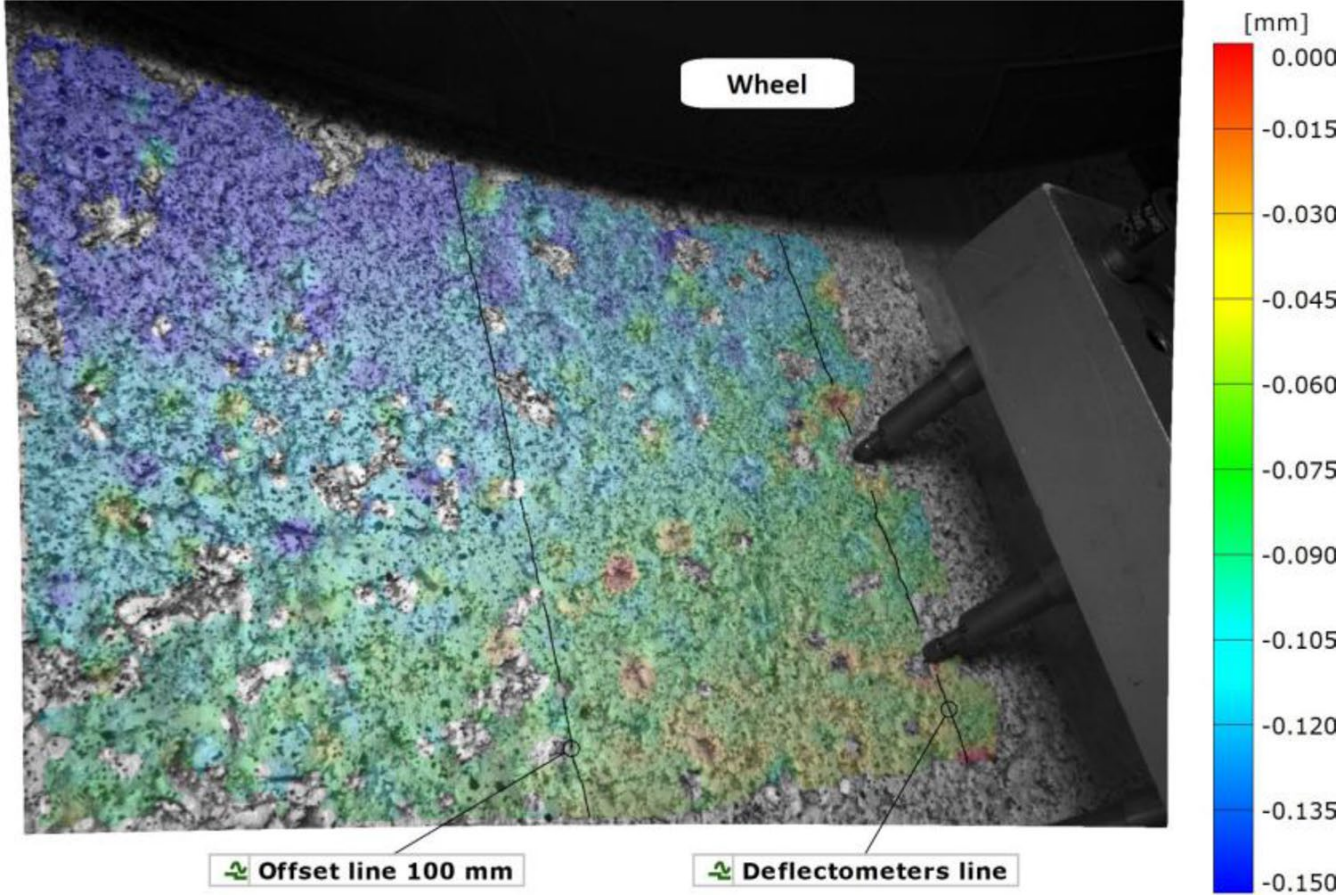
Color map of the deflection basin and seven measuring lines at step 16. Generated with GOM Inspect Pro (Aramis 3D v2019, GOM GmbH, Braunschweig, Germany).

The deflection in the vertical direction is displayed in Fig. 8 along seven curves spaced 30 mm apart. These curves are the intersection between the pavement surface and vertical planes that are parallel to the extensometers.

**Figure 9:**
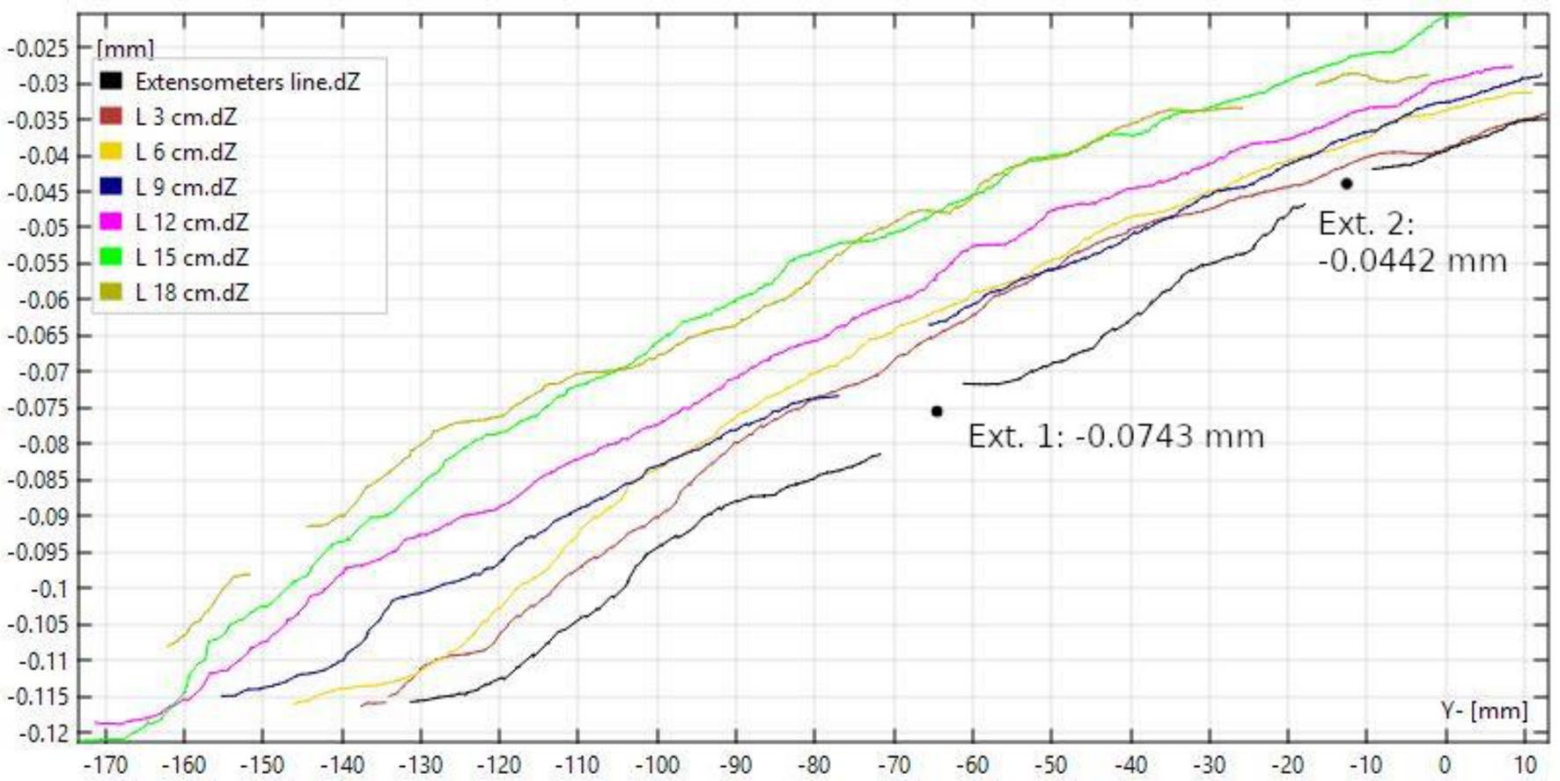
Vertical displacement of the pavement along the sampled lines at step 16.

**Figure 10:**
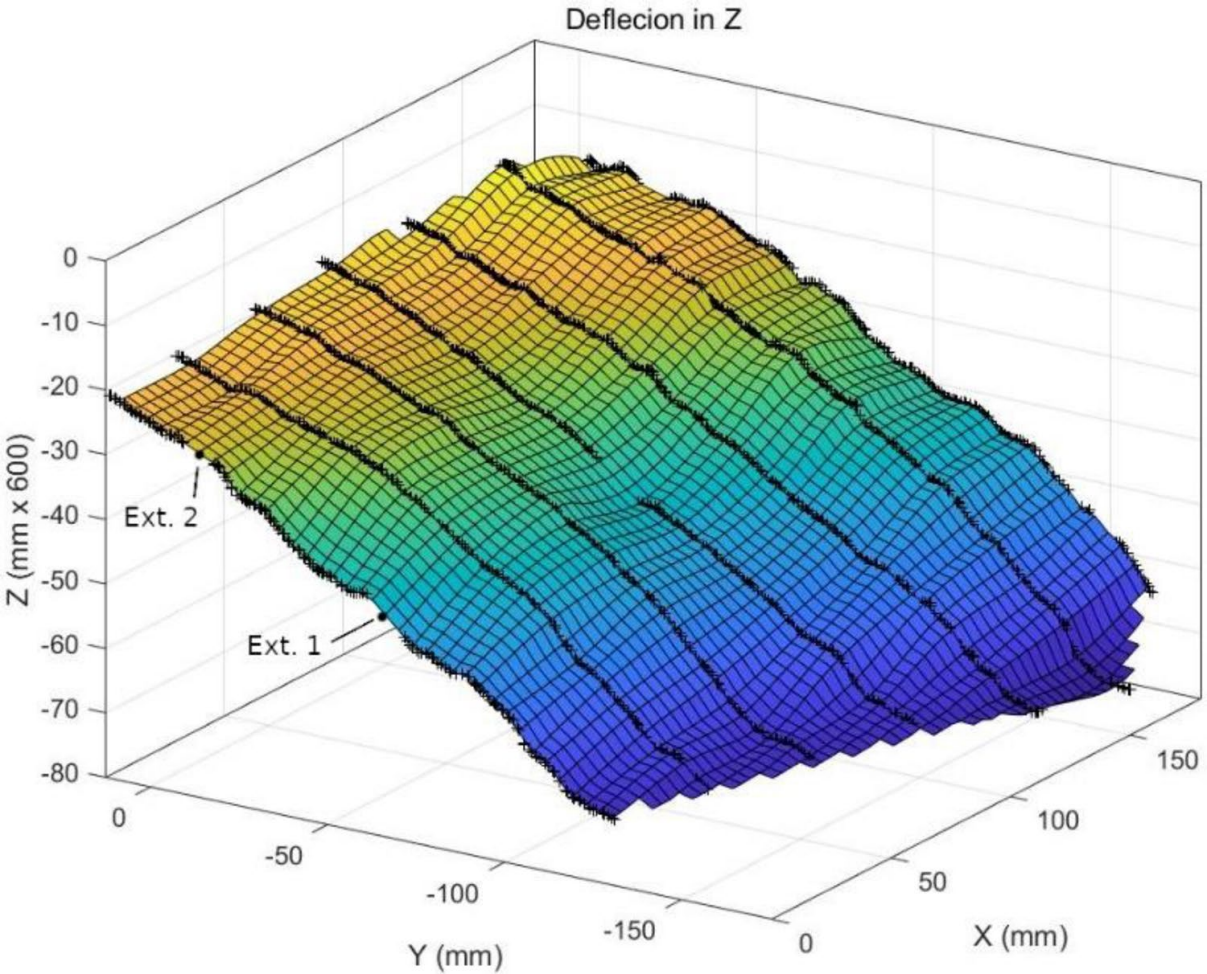
Deformed surface at step 16.

The vertical displacement along the seven curves is shown in Fig. 9 for step 16, which captured the deepest basin. The deflection of the pavement under the sensors was calculated by interpolating the DIC-based curves. A fairly uniform deformation was measured in the fields of view of all the cameras along 180 mm in the wheel motion direction and in the orthogonal direction. Those deflection curves were interpolated to build a 3D surface, which is shown in Fig. 10. From a qualitative point of view, the DIC system performed a realistic capture of the deformation on the pavement.

**Figure 11:**
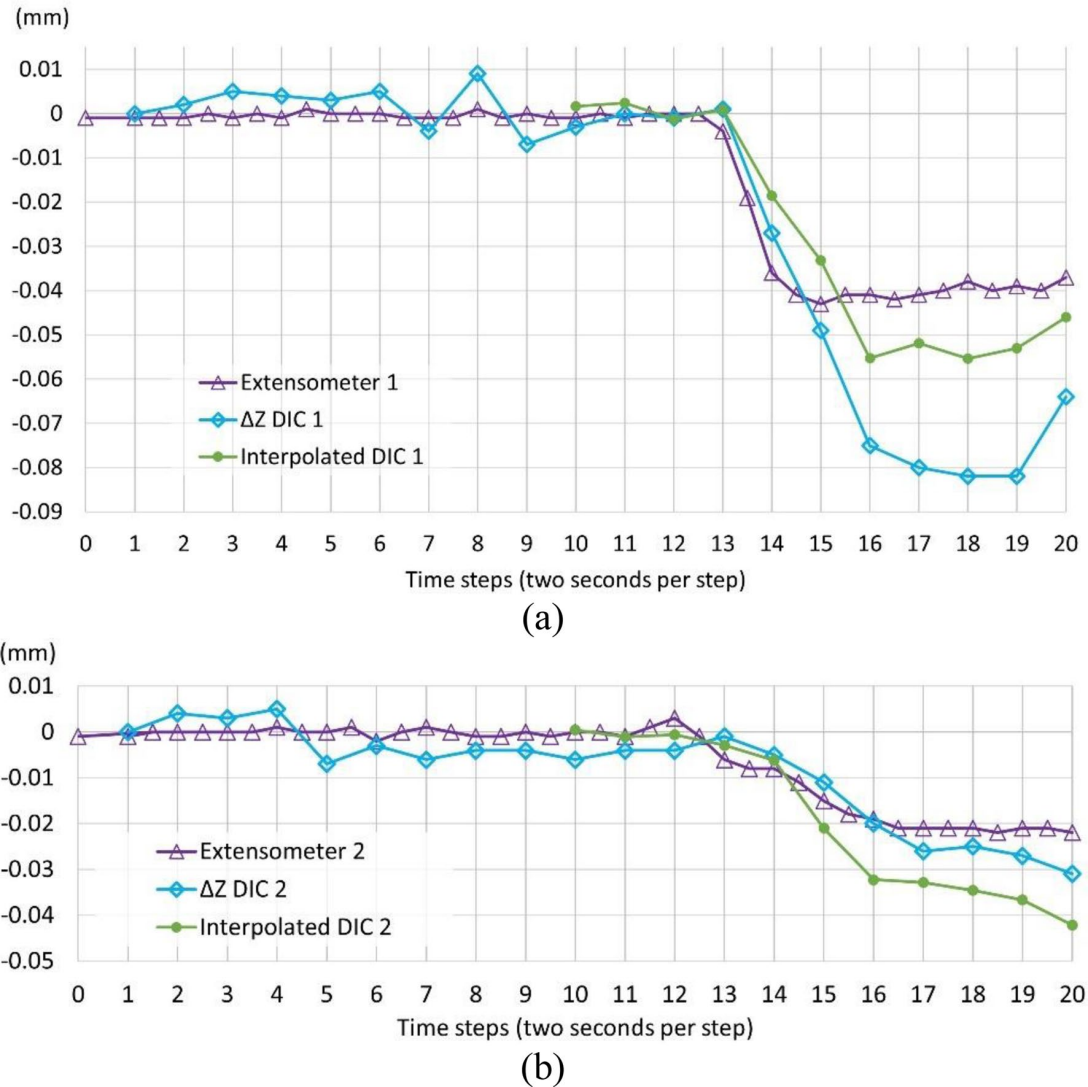
Direct comparison between results from DIC and the extensometer 1 (**a**) and 2 (**b**). The lines represent the cumulative values of: [purple] the Z-displacement from the readings of the contact sensors; [blue] the Z-differences between the fixed and mobile auto-detectable targets placed on sensors from DIC observations (ΔZ DIC); and [green] Z-displacement obtained from the interpolation of DIC-based surface under the contact sensors.

The assessment of the DIC was performed from the deformation detected by DIC on two sets of elements. The first set of elements is the tie points (see Fig. 3). In particular, the difference was calculated between the mobile points and the fixed points of the sensors (ΔZ DIC). The second set of elements is the points of the pavement that were in contact with the sensors. Only the two sensors closest to the wheel pass area (Ext. 1 and Ext. 2) were used in the analysis, as the more distant ones did not register significant variations above their noise-floor during the test. Since the sensors themselves block the visibility of the points of contact from the view of the camera, the deformation values according to DIC were calculated by interpolating the values of the surrounding points (interpolated DIC). Both DIC-based sources of information are compared in Fig. 11 with the results from the extensometers.

The pavement deflection was clearly detected during the tire pass with both the optical and the contact-based methods. However, some variations in readings can be observed along the time steps depending on the measurement method. Regarding to the observations obtained from the speckled surface registered with DIC (green line), one could expect a priori to observe certain differences with the reference contact method (purple line). It should be noted that this comparison entails certain interpolation error due to the visibility of the contact points in the stereo-pair, as explained above. However, with regard to the differences between the displacements of the auto-detectable targets (blue line) and the contact method (purple line), one could expect a closer correspondence, as Aramis 3D is theoretically capable to track the targets with sub-pixel accuracy. A possible cause of this divergence in the last 5 steps of the experiment could lie in a slight displacement of the cameras. A hypothetical explanation is that the deformation basin could have reached the camera supports and caused this small movement. In any case, the order of magnitude of the maximum differences represented in the graphs (few tens of microns) is very close to the precision of the measurement methods themselves, suggesting that the findings should be interpreted cautiously.

In the first contact sensor, from steps 1 to 11, the fluctuations of the positions of the tie points were more pronounced than those from the contact sensors. According to these positions in the Z-direction reported by the targets attached to both contact sensors, the cause could lie in the displacement of the cameras: the deformation basin could have reached the camera supports and caused the cameras to move during the test, since deflection was measured on the fixed tie points. Rigid-body motion of the stereo-camera pair can be corrected in post-processing if there is a fixed reference point somewhere in their field of view^83^. Additional measures can be tested to increase the reliability of the test: searching either a more effective speckle pattern^7^ or a road pavement with an easier-to-process natural speckle pattern^84^. A shaded surface can be also captured^37^, instead of direct natural or artificial lighting.

The deformation values in the vertical direction for steps 10–20 are presented in Table 1. The agreement between the DIC and contact sensors was better for the second sensor due to its lower readings (due to longer distance from the loaded area): The calculated errors had an accuracy (mean value) and a precision (standard deviation) of − 0.016 mm and 0.021 mm for the first sensor, and − 0.002 mm 0.005 mm for the second sensor, respectively.

**Table 1:** Comparison of the deformation according to the sensors and the targets.

Step	ΔZ DIC 1 (mm)	Sensor 1 (mm)	Error 1 (mm)	Absolute error 1 (mm)	ΔZ DIC 2 (mm)	Sensor 2 (mm)	Error 2 (mm)	Absolute error 2 (mm)
10	− 0.003	− 0.001	− 0.002	0.002	− 0.006	0	− 0.006	0.006
11	0.000	− 0.001	0.001	0.001	− 0.004	− 0.001	− 0.003	0.003
12	− 0.001	0	− 0.001	0.001	− 0.004	0.003	− 0.007	0.007
13	0.001	− 0.004	0.005	0.005	− 0.001	− 0.006	0.005	0.005
14	− 0.027	− 0.036	0.009	0.009	− 0.005	− 0.008	0.003	0.003
15	− 0.049	− 0.043	− 0.006	0.006	− 0.011	− 0.015	0.004	0.004
16	− 0.075	− 0.041	− 0.034	0.034	− 0.020	− 0.019	− 0.001	0.001
17	− 0.08	− 0.041	− 0.039	0.039	− 0.026	− 0.021	− 0.005	0.005
18	− 0.082	− 0.038	− 0.044	0.044	− 0.025	− 0.021	− 0.004	0.004
19	− 0.082	− 0.039	− 0.043	0.043	− 0.027	− 0.021	− 0.006	0.006
20	− 0.064	− 0.037	− 0.027	0.027	− 0.031	− 0.022	− 0.009	0.009
Mean value			− 0.016	0.019			− 0.003	0.005
Standard deviation			0.021	0.018			0.005	0.002
Minimum value			− 0.044	0.001			− 0.009	0.001
Maximum value			0.009	0.044			0.005	0.009

In view of the displacement detected by the DIC in the targets of the fixed parts of the sensors, those displacements were subtracted from the interpolated displacement on the pavement detected by the DIC system. This correction was already done in the interpolated DIC values in Fig. 11. These interpolated values derived from the DIC under the contact sensors had more uniform behavior for both sensors; in the first loaded steps, the DIC values were lower than the sensor readings, while the opposite trend was found in the next steps. The precise values and the corresponding absolute errors are shown in Table 2.

**Table 2:** Comparison between the deformation according to the sensors and the DIC-based interpolation.

Step	Interpolated DIC (mm)	Sensor 1 (mm)	Error 1 (mm)	Interpolated DIC (mm)	Sensor 2 (mm)	Error 2 (mm)
11	0.002	0	0.002	− 0.006	− 0.004	− 0.002
12	− 0.001	− 0.001	0	− 0.004	− 0.004	0.000
13	0.001	0.001	0	− 0.019	− 0.001	− 0.018
14	− 0.019	− 0.027	0.008	− 0.018	− 0.005	− 0.013
15	− 0.033	− 0.049	0.016	− 0.034	− 0.011	− 0.023
16	− 0.055	− 0.075	0.020	− 0.044	− 0.020	− 0.024
17	− 0.052	− 0.080	0.028	− 0.046	− 0.026	− 0.020
18	− 0.055	− 0.082	0.027	− 0.036	− 0.025	− 0.011
19	− 0.053	− 0.082	0.029	− 0.022	− 0.027	0.005
20	− 0.046	− 0.064	0.018	− 0.019	− 0.031	0.012
Mean value			0.015			− 0.009
Standard deviation			0.011			0.013
Minimum absolute error			0			− 0.024
Maximum absolute error			0.029			0.012

The error of the DIC system in relation to the extensometers was smaller for the first sensor, which was closest to the loaded pavement. The DIC had an accuracy (mean error) of 0.015 mm with a precision (standard deviation) of 0.011 mm. Thus, the DIC system provided a lower accuracy than its precision. This aligns with the hypothesis about the deformation basin affecting the supports of the DIC cameras. Regarding the second sensor, led to a lower mean error (− 0.009 mm), with a standard deviation of 0.013 mm, respectively.

The correction of the DIC-based deformation under the extensometers by subtracting the deformation detected on their fixed parts led to better agreement between the DIC and sensors. However, as shown in Fig. 11, both DIC-based sources of information still exhibited irregular behavior. Therefore, apart from improving the stability of the cameras stand, a deeper analysis of the other variables should be performed to find the source of error:

- Software processing, including facet size, distance between facet centroids, and spatial and temporal interpolation.
- The optimal speckle pattern on the pavement, as well as the control of natural light on the monitored area. It could be easier to control these conditions under the vehicle. The fluctuations observed on the deformed surface could be related to the fluctuations in this variable.

Previous in-field applications of DIC in pavement analysis are still scarce and most of them do not provide a true accuracy analysis, benchmarking the results with independent techniques. The experiment we carried out, demonstrates that optical methods based on stereo-DIC processing can achieve, even outside the lab, high levels of accuracy in measuring pavement deflections, reaching mean values of absolute errors of 0.019 mm (Table 1). Devices for continuous pavement deflection profiling are currently widely used in many projects, because of their performance. However, some of these techniques using moving vehicular loads such as TSD^85–87^, RDT^88,89^ or RWD^90,91^ generally provide much poorer accuracies (70–250 µm) than those achieved by DIC. Among the most frequently used methods for the continuous pavement deflection profiling, RDD is considered to be one of the most accurate, as employs contact-type loading (with a sinusoidal loading). Some previous studies^92–95^, employing RDD reported errors comparable to, or even lower than, those provided in this test by Aramis 3D. Nevertheless, the current operational speed of RDD is limited to 2 km/h, which also complicates its use in large-coverage projects. To sum up, the results presented in our study are comparable in terms of precision to the conventional methods operated in stationary mode (e.g., FWD) or at slow test speed (e.g., RDD) but clearly outperform the moving load methods with operational speeds.

Indeed, 3D-DIC still has a long way before reaching a massive implementation comparable to that of devices with road vehicle operational speeds^96,97^. However, in isolated sampling applications, in experimental research, or in pavements with complex stress state, DIC could be very helpful, providing accurate measurements of displacements and the entire (3D) deflection basin shape. A good example of a specific application in the diagnostics of existing road pavements could be the case of paving blocks or slabs. Due to high stiffness and confined ability of cooperation of surrounding block elements, in that type of pavement fatigue life is strongly connected with displacement distribution^37^. In any case, further research should be conducted to explore with DIC the extraction of potential indicators from the deflection basin of asphalt pavements to assess their current level of deterioration and carry out timely maintenance or rehabilitation.

## Conclusions

The deformation of asphalt pavement was studied through a high-resolution 3D-DIC system. It could have the potential to reach the level of precision in field tests that is obtained in laboratory tests. The data provided by the DIC system were compared with the readings of extensometers (contact sensors) with ± 1 µm precision, which were adopted as the reference. Their readings were consistent with the viscoelastic behavior of asphalt pavement.

The data obtained by contact sensors matched the expected deformation shape in pavements under a vehicle wheel, and thus, we assume that this data source was an adequate reference. The basin was successfully captured by DIC equipment. The cameras recorded not only the pavement next to the tire but also parts of the pavement that were in front and behind the tire. From a qualitative point of view, 3D-DIC proved suitable for on-site measurements.

In relation to the deflection values, the values provided by the DIC system on the speckle pattern under the sensors (obtained by interpolation) had an accuracy (mean error) of 0.015 mm and a precision (standard deviation) of 0.011 mm. The values provided by the DIC system had in the second sensor higher accuracy and lower precision due to the larger distance between the loaded area, compared to the first sensor.

3D-DIC proved its capability for on-site measuring of deformations on pavements, but the need to refine the methodology was clear. The most evident weakness of the test was the presumed fall of the camera supports within the deformation basin, which affected the DIC measures. More attention should be given to the control of the speckle pattern and light conditions in future tests. Errors introduced by both variables could be limited by placing the cameras under the vehicle. Using high-speed cameras could allow us to measure deformations induced by high-impact loading. Laboratory tests continue to produce better results than in situ tests^98^.

Thus, combining consumer-grade cameras with open-source software could facilitate bringing laboratory precision to on-site measurements from vehicles in motion, not only for 3D-DIC but also to deploy the less costly 2D-DIC in this field of measurement. This particular application is still very scarce in the scientific literature. We gained experience in pavement assessment with this technology. Solving the variables that affect the effectivity of the DIC system, it would be applied on static load-based measuring methods. Installing high-speed cameras, it could be tested in deflection measurement based on stationary dynamic impact. Once the DIC system were well configured, the main challenge will be to operate on a vehicle in motion for a moving dynamic deflection measurement.
